# The Protective Role of the Carbohydrate Response Element Binding Protein in the Liver: The Metabolite Perspective

**DOI:** 10.3389/fendo.2020.594041

**Published:** 2020-11-17

**Authors:** Loranne Agius, Shruti S. Chachra, Brian E. Ford

**Affiliations:** Biosciences Institute, Newcastle University, The Medical School, Newcastle upon Tyne, United Kingdom

**Keywords:** ChREBP, fructose, glucokinase activator, ATP, AMPK, *G6pc*, *Pklr*, *Gckr*

## Abstract

The Carbohydrate response element binding protein, ChREBP encoded by the *MLXIPL* gene, is a transcription factor that is expressed at high levels in the liver and has a prominent function during consumption of high-carbohydrate diets. ChREBP is activated by raised cellular levels of phosphate ester intermediates of glycolysis, gluconeogenesis and the pentose phosphate pathway. Its target genes include a wide range of enzymes and regulatory proteins, including *G6pc*, *Gckr*, *Pklr, Prkaa1,2*, and enzymes of lipogenesis. ChREBP activation cumulatively promotes increased disposal of phosphate ester intermediates to glucose, *via* glucose 6-phosphatase or to pyruvate *via* glycolysis with further metabolism by lipogenesis. Dietary fructose is metabolized in both the intestine and the liver and is more lipogenic than glucose. It also induces greater elevation in phosphate ester intermediates than glucose, and at high concentrations causes transient depletion of inorganic phosphate, compromised ATP homeostasis and degradation of adenine nucleotides to uric acid. ChREBP deficiency predisposes to fructose intolerance and compromised cellular phosphate ester and ATP homeostasis and thereby markedly aggravates the changes in metabolite levels caused by dietary fructose. The recent evidence that high fructose intake causes more severe hepatocyte damage in ChREBP-deficient models confirms the crucial protective role for ChREBP in maintaining intracellular phosphate homeostasis. The improved ATP homeostasis in hepatocytes isolated from mice after chronic activation of ChREBP with a glucokinase activator supports the role of ChREBP in the control of intracellular homeostasis. It is hypothesized that drugs that activate ChREBP confer a protective role in the liver particularly in compromised metabolic states.

## ChREBP—A Transcription Factor Activated by Dietary Carbohydrate

The liver has a central role in carbohydrate metabolism by net production of glucose by glycogenolysis and gluconeogenesis in the post absorptive state to maintain blood glucose homeostasis and net uptake of dietary sugars after a meal when the products of dietary carbohydrate digestion comprising glucose, fructose and other sugars are absorbed from the intestine into the portal vein and transported directly to the liver. The Carbohydrate response element binding protein ChREBP, is a transcription factor that is adaptively expressed in the liver in response to high dietary carbohydrate loads and it is also expressed in the intestine, kidney, adipose tissue, and pancreatic β-cells (1–3). It was first purified from livers of rats fed a high carbohydrate diet in a search for the mechanism by which the liver pyruvate kinase gene (*Pklr*) is induced by high glucose (1). Prior work had identified several genes including *Pklr* and enzymes of lipogenesis that are induced in hepatocytes by high glucose, independently of insulin through a consensus sequence (ChoRE, carbohydrate response element) composed of two enhancer (E)-boxes (CANNTG) separated by five nucleotides (4, 5). ChREBP is commonly described as a “lipogenic transcription factor” that mediates the conversion of glucose into lipid in liver, adipose tissue, and pancreatic β-cells or as a “glucose-sensor” because it is activated in liver and pancreatic β-cells in response to elevated glucose (6, 7). Genome-wide analysis of ChREBP binding sites in mouse liver and white adipose tissue identified thousands of candidate target genes, some of which are consistent with a lipogenic role whereas others, which remain to be functionally validated, implicate more diverse functions (8). One proposed function for ChREBP is in maintenance of cellular ATP and metabolite homeostasis (9, 10). Although this function is expected to be ubiquitous, it has a particularly important role in the liver which is exposed to a wider and more variable concentration range of glucose and fructose than extrahepatic tissues, because the products of carbohydrate digestion that are absorbed from the gut are delivered directly to the liver *via* the portal vein. Here we review recent evidence for the role of ChREBP in metabolite homeostasis in liver.

## ChREBP Structure and Isoforms

ChREBP, also known as MondoB, and its close paralog MondoA, which is expressed at high levels in muscle, are members of the Myc-Mlx superfamily of basic helix-loop-helix leucine zipper transcription factors. Both ChREBP and MondoA bind to the DNA E-boxes by forming heterodimers with Mlx (Max like protein X). Accordingly, the gene names of MondoA and ChREBP are *Mlxip* (Mlx interacting protein) and *Mlxipl* (Mlx interacting protein like), respectively (11).

ChREBP is a large ~100 kDa protein comprising a DNA binding domain in the C-terminal region and nuclear import and export signals in the N-terminal region which interact with importins and the scaffolding protein 14-3-3 during shuttling between the cytoplasm and nucleus. The N-terminal segment contains regions conserved in Mondo homologs that comprise a low glucose inhibitory domain (LID) that controls a glucose response activation conserved element (GRACE) which mediates transcriptional activation (12). ChREBP (full-length) translocates at high glucose from the cytoplasm to the nucleus and conformational changes in the LID and GRACE modules allow its binding to the ChoRE elements on target genes and recruitment of co-regulators. This model was supported by a truncated form of ChREBP lacking the N-terminal 1-196 residues (containing the inhibitory domain) which is constitutively active at low glucose (13, 14). For detailed reviews see (6, 7).

A key breakthrough in understanding ChREBP function came with the identification by Herman and colleagues of a second shorter isoform, termed ChREBP-β, resulting from alternative splicing at a new exon-1B, with a different promoter and transcription start site at exon-4 resulting in a shorter protein (687 *vs.* 864 amino acids) than the full-length ChREBP which is now called ChREBP-α (15). The β-isoform shows some functional similarities to the truncated ChREBP lacking the autoinhibitory domain (667 amino acids) in being constitutively nuclear and thereby fully active at low glucose (15). This contrasts with ChREBP-α which is predominantly present in the cytoplasm and translocates to the nucleus only during metabolite challenge (16). The ChREBP-β isoform was first identified in adipose tissue but later confirmed to be expressed in liver, intestine, kidney and pancreatic β-cells though not in skeletal muscle (17–20). Intriguingly, a comparison of the functional ChoRE sites of ChREBP-β in adipocytes, liver and pancreatic β-cells found that liver and β-cells share a common ChoRE element that is further upstream from the ChoRE that is active in adipocytes and adipose tissue (19), indicating tissue-specific transcriptional regulation of ChREBP-β, but also similarities between liver and pancreatic β-cells.

## ChREBP-β mRNA—A Biomarker of ChREBP Activation

The expression of ChREBP-β is driven by a ChoRE and accordingly by substrate-mediated translocation of ChREBP-α into the nucleus (15, 19). What has been more challenging to unpick is the role of ChREBP-β in auto-regulation of ChREBP-α and ChREBP-β (19, 20) and also the relative roles of ChREBP-α and ChREBP-β on the downstream target genes (8, 21).

Prior to the discovery of the ChREBP-β isoform, ChREBP mRNA levels in liver or isolated hepatocytes were found to be modestly (< 2-fold) raised in conditions of markedly elevated glucose 6-P as occurs in experimental models of high glucose or glucose 6-phosphatase deficiency (22). However, selective measurement of ChREBP-β versus ChREBP-α mRNA showed in most cases greater fold-changes in ChREBP-β than ChREBP-α during metabolic activation in adipose tissue, liver, intestine, and pancreatic β-cells. In mouse adipose tissue ChREBP-β was more responsive to overnight fasting and refeeding and to overexpression or downregulation of Glut4 (15). In mouse liver, dietary glucose or fructose and glucokinase activator (GKA) drugs caused 4-8-fold elevation in ChREBP-β mRNA with little change in ChREBP-α mRNA, with similar responses in mouse hepatocytes challenged with sugars and GKAs (23, 24). Interestingly in rats, fasting and refeeding caused a 40-fold increase in ChREBP-β (25). It is noteworthy that *Gck* mRNA also shows several-fold larger changes in rat compared with mouse hepatocytes (24, 26, 27) but whether this is due to intrinsic species differences or to differences in the severity of nutritional state remains unclear.

Two elegant complementary studies on the ChREBP-β isoform in pancreatic β-cells using si-RNA to selectively target the ChREBP-β transcript, showed that the latter isoform mediates glucose-induced β-cell proliferation and induction of ChREBP target genes (19) but it also exerts negative feedback on ChREBP-α mRNA and on downstream ChREBP target genes (20). These studies also showed a remarkably wide variability in the ChREBP-β to ChREBP-α mRNA ratio as a function of extracellular glucose concentration in islet and β-cell models (19) and in various mouse models of diabetes (20). Zhang and colleagues (19) showed ~8-fold lower ChREBP-β to ChREBP-α in the proliferating rat INS-1E β-cell line but far lower ratios (> 1000–10,000 fold) in rat and human islets. Correspondingly, the fold increment in ChREBP-β mRNA during glucose challenge paralleled the fractional initial value ranging from 8-fold to 1000-fold for the β-cell line and islets, respectively.

**Figure 1:**
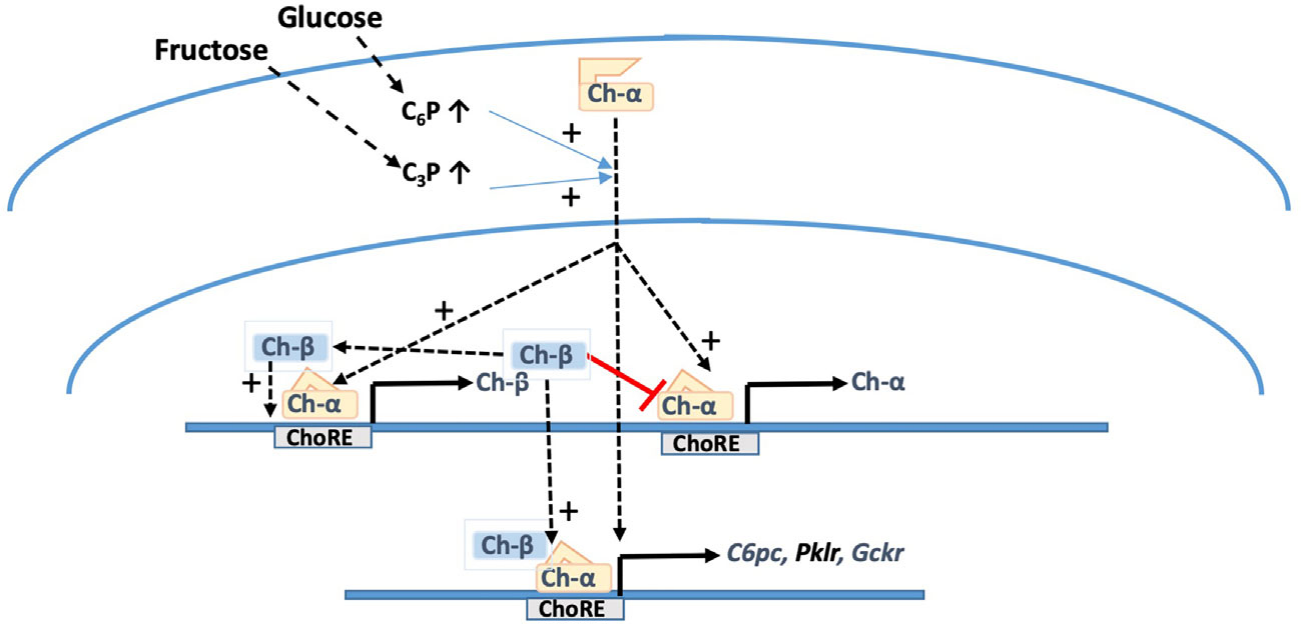
Metabolite-mediated activation of ChREBP-α and induction of ChREBP-β. Substrates that raise cellular levels of hexose-phosphates (C_6_P) and triose-phosphates (C_3_P) cause translocation of full-length ChREBP-α from the cytoplasm to the nucleus and binding to upstream (ChREBP-β) and downstream (ChREBP-α) ChoREs of the ChREBP gene and to ChoREs of various ChREBP target genes *G6pc*, *Pklr*, and *Gckr*. ChREBP-β which unlike ChREBP-α is present constitutively in the nucleus exerts positive feedback on its own promoter but negative feedback on the downstream ChREBP-α promoter. This accounts for the modest changes in ChREBP-α mRNA levels in comparison with ChREBP-β mRNA during high glucose activation.

The expression of ChREBP-β is driven by both ChREBP-α which is activated by high glucose and by ChREBP-β itself (which is constitutively nuclear and active) through a positive feed-back loop resulting in a sustained increase in ChREBP-β mRNA in conditions of high glucose (19). The elevated ChREBP-β protein in turn also causes repression of ChREBP-α at mRNA and protein levels and also of downstream target gene expression (*Pklr, Txnip, Acc1*) (20). This implicates a role for the ChREBP-α to ChREBP-β ratio in the regulation of downstream target genes and it also supports a role for raised ChREBP-β mRNA levels as a marker of ChREBP-α activation (**Figure 1**). In human adipose tissue and liver, ChREBP-β mRNA levels correlate with the expression of ChREBP target genes (15, 28, 29), and in adipose tissue ChREBP-β levels also correlate with insulin sensitivity (28, 29) whereas in liver raised ChREBP expression correlates with insulin resistance (29), most likely because ChREBP target genes include *G6pc* and *Gckr* (23, 27, 30), and induction of these genes predicts impaired hepatic glucose clearance (9).

## The Metabolite Signal for ChREBP-α Translocation to the Nucleus and Activation

### The Case for Glucose 6-P and the Inefficacy of 2-Deoxyglucose 6-P

ChREBP-α translocates to the nucleus in conditions of high glucose (16). However, the molecular signaling events are not resolved. Glucose *per se* is ineffective because mannoheptulose and 5-thioglucose which inhibit glucose phosphorylation block the induction of ChREBP target genes by high glucose (5, 27, 30). Inhibitors of glucose 6-phosphatase which markedly raise phosphate ester intermediates (30) implicate a mechanism linked to raised intracellular metabolites rather than metabolic flux, although the latter cannot be unequivocally excluded. Although these studies show a correlation with elevated glucose 6-P, linked metabolites such as fructose 6-P, fructose 2,6-P_2_, and downstream intermediates of glycolysis cannot be excluded (30, 31). A role for glucose 6-P in ChREBP activation is widely inferred based on bioinformatic (32) and other considerations (33, 34). However, the glucose analogue 2-deoxyglucose, which is phosphorylated on the 6-position by glucokinase but is not further metabolized by glycolysis, has modest effects on ChREBP activation in proliferating β-cells compared with high glucose (34, 35), and is totally ineffective in parenchymal hepatocytes (5, 30, 31), despite accumulating to high intracellular levels and particularly when combined with an inhibitor of glucose 6-phosphatase (30, 31). This total lack of efficacy of 2-deoxyglucose on ChREBP target gene induction in hepatocytes rules out an exclusive role for glucose 6-P although it does exclude a contributory role in conjunction with other metabolites or covalent modification mechanisms.

### Xylulose 5-P and Fructose 2,6-Bisphosphate

The induction of ChREBP target genes including *Pklr* and *G6pc* in cell models is not confined to high glucose, but other substrates that bypass glucokinase such as xylitol, dihydroxyacetone and fructose can mimic high glucose, implicating other candidate metabolites (30). Uyeda and colleagues identified xylulose 5-P as an activator of a type 2A protein phosphatase that dephosphorylates ChREBP and proposed that raised xylulose 5-P in conditions of high glucose activates ChREBP through dephosphorylation (36). Other studies showed that dephosphorylation alone is insufficient for high-glucose activation (33). The xylulose 5-P sensitive phosphatase also dephosphorylates the bifunctional enzyme PFKFB1 (phosphofructokinase-2/fructose bisphosphatase-2) that generates and degrades the signaling metabolite, fructose 2,6-P_2_ (37). This is synthesized by PFKFB1 from fructose 6-P and its levels in hepatocytes correlate with hexose 6-P in conditions of basal cAMP (30, 38). In conditions of raised glucagon levels, protein kinase A mediated phosphorylation of Ser32 inhibits the kinase activity and PFKFB1 thereby functions as a bisphosphatase converting fructose 2,6-P_2_ to fructose 6-P. Expression of a kinase-deficient bisphosphatase active variant of PFKFB1 to deplete fructose 2,6-P_2_ abolishes ChREBP activation in conditions of high glucose or gluconeogenic precursors, implicating an essential role for raised fructose 2,6-P_2_ in ChREBP activation (30, 38). This indicates an additional role of xylulose 5-P in ChREBP activation through raised fructose 2,6-P_2_ (37). Unlike intermediates of the glycolytic pathway which cannot be modulated independently of proximal or distal metabolites (31), fructose 2,6-P_2_ can be modulated more selectively because it is a dead-end metabolite, derived from and degraded to fructose 6-P. It is noteworthy, however, that fructose 2,6-P_2_ could only be modulated in conditions of raised glucose 6-P or triose phosphates and therefore additional co-ordinate roles for glucose 6-P or other metabolites cannot be excluded (30, 38).

### Inhibitory Metabolites Favoring ChREBP Sequestration in the Cytoplasm

Various metabolites have been identified that promote sequestration of ChREBP in the cytoplasm including the ketone bodies, 3-hydroxybutyrate, and acetoacetate which are elevated during fatty acid mobilization from adipose tissue (39) and raised AMP levels, which occur in liver during fasting in conjunction with a decrease in the ATP/ADP ratio (40). AMP was shown to bind to the bimolecular complex of ChREBP and the targeting protein 14-3-3 but not to either protein in isolation and may thereby favor sequestration of ChREBP bound to 14-3-3 in the cytoplasm (40). This effect of raised AMP may in part contribute to the inhibition of recruitment of ChREBP to the nucleus by metformin in conditions of high glucose (41, 42). Cell metformin levels within the therapeutic range inhibit the high-glucose mediated induction of ChREBP target genes, in conjunction with lowering of glucose 6-P and fructose 2,6-P_2_ (41, 42). Accordingly, multiple metabolites including lower glucose 6-P and fructose 2,6-P_2_ and raised AMP may all be involved in the counter-regulatory effect of metformin on the glucose activation. The inhibitory effect of AMP on ChREBP translocation could also explain why in hepatocytes fructose is less effective than high glucose in ChREBP target gene induction (41).

### Covalent Modification of ChREBP: Role for Acetylation in Activation

ChREBP is phosphorylated by protein kinase-A on residues within the N-terminal (S196) region that has the nuclear export and import sequences and within the DNA binding domain (S626, T666). Phosphorylation affects the binding to importin proteins which promote translocation to the nucleus and also promotes binding to 14-3-3 which favors sequestration in the cytoplasm (43). Covalent modification by O-GlcNAcylation promotes stabilization from degradation (44) but does not seem to be involved in activation. Modification by acetylation of Lys672 within the DNA binding domain catalyzed by the histone acetyl transferase coactivator P300 has been implicated in activation of ChREBP in hepatocytes (45). It was proposed that models of insulin resistance characterized by activation of protein kinase-A promote inhibition of the salt-inducible kinase (SIK) which in turn promotes activation of the acetyltransferase P300 enhancing ChREBP acetylation (45).

Acetylation on Ne-lysine residues is of particular interest as a mechanism for ChREBP activation because it is an additional link to metabolite control *via* cellular levels of acetyl-CoA and the NAD^+^/NADH redox state (46). There are 22 lysine acetyltransferases (KAT) in five major families of which P300 has been one of the most intensely studied and there are 18 lysine deacetylases (KDAC) of which the NAD^+^-dependent sirtuins have been the most studied (46). Acetylation is dependent on the level of substrate, acetyl-CoA, and deacetylation on the level of NAD^+^ and thereby on the NAD^+^/NADH redox state. Raised acetyl-CoA is expected in conditions of substrate overload with high glucose, fructose, xylitol or ethanol, all of which are known to cause ChREBP activation (24, 47, 48). Depletion of NAD^+^, occurs with reduced substrates such as xylitol and ethanol and is further enhanced when the malate aspartate shuttle which transfers NADH reducing equivalents from the cytoplasm to the mitochondria is inhibited with amino-oxyacetate (42). In hepatocytes ChREBP is very strongly activated by xylitol in combination with inhibition of malate aspartate shuttle despite modest elevation in hexose phosphates (24). The represents an analogous metabolic state as occurs with ethanol (47, 48) supporting a potential role for acetylation in mediating the effects of other reduced substrates like xylitol.

## Insights into ChREBP Function From ChREBP-Knock Down Models

**Figure 2:**
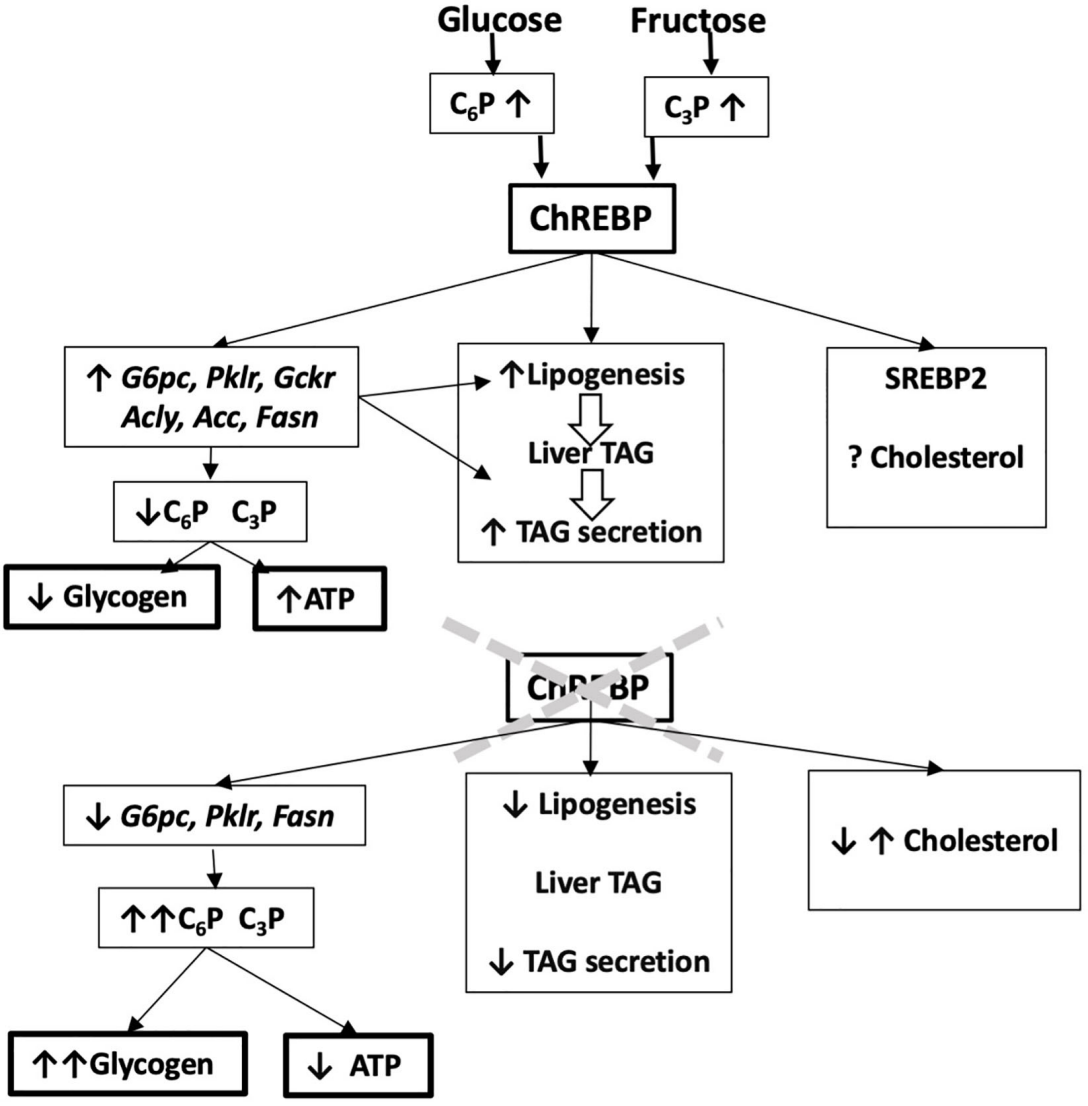
Predicting ChREBP function in liver from ChREBP-knock down models. Liver-selective or global ChREBP deletion mouse models have attenuated mRNA levels of ChREBP target genes, raised hexose phosphate (C_6_P) and triose phosphate (C_3_P) esters, increased glycogen storage and decreased lipogenesis and hepatic triglyceride secretion. They also have impaired hepatic ATP homeostasis. This predicts a role for ChREBP in protecting from liver damage by carbohydrate overload through induction of ChREBP target genes and attenuated glycogen storage and improved phosphate ester and ATP homeostasis.

Genome-wide association studies have identified variants in the *Mlxipl* gene that associate with blood lipids, markers of liver disease and inflammation (49–51). However, there is limited information on how these variants affect ChREBP function. Most of the studies using ChREBP knock-down models have provided evidence for a protective role for ChREBP in liver function (**Figure 2**), particularly during challenge with a fructose-containing diet (52–56) or in other compromised metabolic states such as glucose 6-phosphatase deficiency (57). ChREBP knock-down studies have used either total ChREBP deletion (23, 52, 53, 58) or liver-selective models generated by crossing ChREBP^flox^ mice with albumin-Cre transgenic mice (LiChREBP^-/-^) (17, 54, 55) or by short-term ChREBP knock-down using antisense oligonucleotides (56) or shRNA with adenoviral vectors (57, 59). While the focus of the earlier studies was on the liver, ChREBP is also important in the intestine (17) and in adipose tissue (60). In adipose tissue, ChREBP has a co-ordinate role with the adaptive glucose transporter Glut4 which is activated by insulin leading to increased transport of glucose and phosphorylation to generate glucose 6-P, which is further metabolized by glycolysis and lipogenesis (15). Selective knock-down of ChREBP in adipose tissue results in impaired Glut4 activation by post-transcriptional mechanisms supporting a requirement for ChREBP for enhanced glucose metabolism in adipose tissue (60).

Total-ChREBP^-/-^ models are more susceptible to fructose-induced toxicity than LiChREBP^-/-^ as shown by the high mortality when transferred to diets high in fructose or sucrose (17, 52, 61, 62). This is due to ChREBP deficiency in the intestine which results in compromised fructose absorption, gastrointestinal stress, inflammation, and food aversion (17). The intestine expresses all the enzymes required for fructose metabolism by the “Hers pathway” (63), namely the liver isoform of ketohexokinase (*Khk-C*), aldolase B (*Ald-B*), and triose kinase (*Tkfc*), as well as fructose 1,6-bisphosphatase (*Fbp1*) and glucose 6-phosphatase (*G6pc*) enabling conversion of triose phosphates to glucose (17, 61). The respective genes together with *Slc2a5*, encoding the fructose transporter Glut5, are all induced in the intestine during chronic consumption of high-fructose diets (61). This fructose-induced gene induction is dependent on ChREBP as shown by the blunted induction in global or intestine-selective ChREBP^-/-^ models (17, 61). Whereas wild-type mice respond to diets enriched in fructose or sucrose by increased food intake, the global and intestine-selective ChREBP^-/-^ mice decrease their food intake by up to ~70% compared with ~25% decrease for LiChREBP^-/-^ (17, 61). Strong aversion to dietary fructose also occurs in mouse models of fructose malabsorption due to deficiency in Glut5, ketohexokinase, and triose kinase (64–66).

Key insights into the roles of intestinal fructose metabolism or absorption and food aversion have emerged from studies using stable isotopes combined with knock-down of genes essential for fructose metabolism (66, 67). Jang and colleagues determined intestinal fructose metabolism and absorption, by oral gavage of mice with equimolar loads of ^13^C-labeled fructose and glucose ranging from 0.2 to 2 g/kg body weight and analysis of ^13^C-labeled substrates in the portal vein (67). This showed that intestinal fructose metabolism saturates at ~0.5g/kg body weight, whereas fructose absorption into the portal vein saturates around 2 g/kg body weight. During fructose gavage, fructose 1-P the first intermediate of fructose metabolism accumulates to higher levels in the intestine (> 6 nmol/mg protein) than in liver (67) or in hepatocytes challenged *ex vivo* with high fructose (68). This could be explained by the high fructose concentrations in the gut lumen and the high capacity of the intestinal epithelium for uptake of fructose (*via* Glut*5*) and inorganic phosphate (64, 69), whereas fructose metabolism in hepatocytes is limited by the capacity of the fructose transporter (70) and by hepatic uptake of inorganic phosphate (Pi), as shown by the acute depletion of liver cytoplasmic Pi which in turn leads to compromised mitochondrial ATP production (71). At low doses of dietary fructose that do not exceed the intestinal capacity for fructose metabolism (< 0.5 g/kg in rodent equivalent to 3 g per person in man (67)) the exposure of the liver to fructose is in the micromolar range (< 0.2 mM) (67). Fructose concentrations of 50–200 µM do not cause hepatic ATP depletion (72) but can maximally activate liver glucokinase by dissociating it from the glucokinase regulatory protein resulting in stimulation of glycogen synthesis and glycolysis (73, 74). Estimates of sugar consumption in man range from 8%-20% by energy or 30–100 g sucrose/per day (75). If intestinal fructose metabolism in man saturates at equivalent levels as in rodents, when normalized for energy intake (67), then Western diets with a high sugar intake (75) would exceed the capacity for intestinal fructose metabolism resulting in liver exposure to millimolar fructose, which causes ATP depletion (71).

Studies on LiChREBP^-/-^ models from three independent groups have shown that during challenge with high-fructose or high-sucrose diets the ChREBP deficiency in liver associates with raised plasma alanine aminotransferase (ALAT) activity, a marker of hepatocyte damage, establishing a protective role for hepatic ChREBP in fructose metabolism (17, 54, 55). Various hypotheses can be considered for ChREBP-mediated protection from fructose-induced liver damage. One proposed hypothesis is that ChREBP attenuates cholesterol biosynthesis by promoting SREBP2 degradation (53). This was supported by raised liver cholesterol levels in a global-ChREBP^-/-^ model and lowering of cholesterol by ChREBP overexpression (53). However in LiChREBP^-/-^ models on high-fructose diets, liver cholesterol was either decreased or unchanged despite raised ALAT (17, 54, 55). Raised hepatic cholesterol in global-ChREBP^-/-^ (53), may be linked to endotoxemia consequent to intestinal dysfunction and inflammation as was observed in intestinal-ChREBP*^-/-^* mice (17). A further hypothesis is that ChREBP attenuates accumulation of hepatic triglycerides and diacylglycerides by promoting triglyceride secretion (54, 56, 57). However, the raised ALAT in the LiChREBP^-/-^ did not associate with raised liver triglycerides or diacylglycerides (17, 54–56), implicating mechanisms other than cholesterol, triglyceride or diacylglyceride accumulation in the ChREBP-mediated protection.

One consistent finding on liver metabolic intermediates from ChREBP-deficient models irrespective of whether global, liver-selective or short-term repression is raised phosphate ester intermediates of glycolysis including glucose 6-P and phosphoenolpyruvate (17, 55, 58, 59) and of the pentose phosphate pathway (57) but not UDP-glucose (17, 55) or fructose 2,6-P_2_ (58). The target genes of ChREBP include *G6pc* (23, 30) and the pentose phosphate pathway enzymes (8). The raised levels of the substrates of these enzymes are consistent with a role for ChREBP in maintaining cellular homeostasis of the intermediates (10). One candidate link to the raised phosphate esters is enhanced glycogen storage through allosteric activation of glycogen synthase (76) and inactivation of glycogen phosphorylase by the raised glucose 6-P levels (77). Raised liver glycogen levels in ChREBP knock-down models have been shown on both starch-containing and fructose-containing diets (22, 23, 55, 58, 59) and a role for excessive glycogen accumulation in hepatoxicity was inferred from the correlation between hepatic glycogen levels and raised ALAT in a LiChREBP^-/-^ model on a high-fructose diet (55).

Another mechanism linked to compromised phosphate ester homeostasis particularly during fructose challenge which results in rapid elevation in fructose 1-P (23), is the acute lowering of hepatic ATP (71, 72). This is explained by sequestration of phosphate in fructose 1-P and triose phosphates, depleting inorganic Pi (78), which is a substrate for mitochondrial oxidative phosphorylation, resulting in a decrease in the ATP/ADP ratio and raised AMP through the adenylate kinase equilibrium. The raised AMP is degraded to inosine and then to uric acid (72). In a short-term ChREBP knock-down rat model, fructose feeding was associated with raised plasma uric acid (56) implicating compromised hepatic ATP homeostasis (72) in ChREBP deficiency. It is noteworthy that in rodents as in most species other than primates that lack uric acid oxidase, the uric acid is further metabolized to allantoin and elevation in hepatic urate production may not be apparent from plasma urate levels (72). Compromised ATP homeostasis also occurs in global ChREBP^-/-^ mice in conjunction with a reduced cytoplasmic NADH/NAD redox state (52) and in LiChREBP^-/-^ mice on either a control or a high-fructose diet (55), and this effect was reversed by *Pklr* overexpression implicating a role for this ChREBP target gene in phosphate homeostasis (55).

Production of reactive oxygen species is implicated in the hepatic dysfunction linked to high-fructose diets (66). During fructose metabolism, glyceraldehyde generated from fructose 1-P by aldolase B cleavage can be metabolized by either of three enzymes: triose kinase (*Tkfc*) which generates glyceraldehyde 3-P; aldehyde dehydrogenase (Aldh) which oxidizes glyceraldehyde to glycerate; and alcohol dehydrogenase (Adh) which generates glycerol (63). Based on the lower affinity of Aldh and Adh for glyceraldehyde compared with triose kinase, Sillero and colleagues (63) proposed a hierarchy of mechanisms whereby triose kinase has a predominant role at low glyceraldehyde whereas Aldh and Adh have more prominent roles at raised glyceraldehyde when triose kinase becomes rate limiting. Liu and colleagues showed that selective deletion of triose kinase in liver causes decreased partitioning of fructose to lipogenesis and increased production of glycerate and aggravated oxidative stress and inflammation (66). They proposed a protective role for triose kinase by restraining oxidative stress and favoring lipogenesis (66).

## The Fructose and Glucokinase Activator Paradoxes

The increase in prevalence of non-alcoholic fatty liver disease (NAFLD) and other components of metabolic syndrome including raised blood triglycerides, uric acid and markers of inflammation is attributed to the increased consumption of fructose in the diet (79). Therefore, better understanding of the mechanisms by which ChREBP protects from fructose-induced liver damage can advance the development of new therapies for NAFLD. A key feature of fructose metabolism in respect of ChREBP regulation is that *Slc2a5*, which encodes Glut5, the fructose transporter, and also *Khk*, *AldB*, and *Tkfc* which encode the first three enzymes of fructose metabolism are all functional ChREBP target genes (54, 80), predicting enhanced hepatic fructose clearance and metabolism during ChREBP activation. This contrasts with hepatic glucose metabolism where the activity of Glut2 (encoded by *Slc2a2*) is not limiting unlike Glut5 in relation to fructose metabolism (70) and furthermore *Gck*, which determines the flux-generating step of glucose disposal by the liver, is not a ChREBP target gene (27). Accordingly ChREBP activation in liver does not increase hepatic glucose clearance but it restores metabolite homeostasis in conditions of high glucose by targeting downstream target genes *Gckr* and *G6pc* (9, 10, 23, 30) which restore metabolite homeostasis without increasing glucose clearance. Given that *Slc2a5*, *Khk*, *AldB*, and *Tkfc* are positively regulated by ChREBP, the protective effect of liver ChREBP in conjunction with dietary fructose (17, 54, 55) seems paradoxical. In man, autosomal recessive loss-of-function mutations in the *KHK* gene resulting in ketohexokinase deficiency, manifest as a benign condition “essential fructosuria” in which fructose is excreted in the urine because of impaired hepatic clearance (81) and likewise in mice *Khk* deletion has negligible phenotype (82) and in conjunction with *AldB* deficiency is protective (83). Loss-of-function mutations in *AldB* cause hereditary fructose intolerance, a severe condition which manifests as acute liver damage with ATP depletion and hyperuricaemia on consumption of fructose, leading to liver failure (84). Two hypotheses can be considered for the protective effect of ChREBP in conjunction with a high-fructose diet. First, that ChREBP deficiency results in greater impairment of *AldB* and *Tkfc* relative to *Khk* and *Slc2a5* thereby mimicking *AldB* or *Tkfc* deficiency (66, 84). Second, that other ChREBP target genes such as *Pklr*, *G6pc* and *Gckr* have an overriding role in the adaptive response to fructose. Support for the latter hypothesis was proposed based on Pklr mediated protection in a Li-ChREBP^-/-^ model (55). A role for triose kinase (*Tkfc*) was supported by the aggravated oxidative stress and inflammation resulting from *Tkfc* knock-down (66).

As discussed above, a key feature of hepatic fructose metabolism that manifests at micromolar fructose is activation of glucokinase by dissociation from the glucokinase regulatory protein encoded by the *Gckr* gene, thereby stimulating hepatic glucose disposal (73, 74). In this context glucokinase activators (GKA) mimic the effect of micromolar fructose (85). Recent work exploring the chronic effects of a glucokinase activator on the liver provided evidence for activation of ChREBP as determined from raised levels of ChREBP-β and for improved ATP homeostasis in the isolated hepatocytes from the mice when challenged ex vivo with either xylitol or high glucose in combination with metabolic inhibitors (24). Paradoxically, improved ATP homeostasis in conditions of high substrate challenge, occurred despite sustained elevation in phosphate esters (24). This implicates mechanisms other than the known target genes (*Pklr*, *G6pc*, *Gckr*) encoding enzymes or regulatory proteins of the glycolytic and gluconeogenic pathway. Genome wide analysis had identified, two of the subunits of AMPK as candidate target genes of ChREBP (8). Compromised ATP homeostasis in hepatocytes from AMPK-deficient mice is well-documented (42, 86), making AMPK a candidate functional target of ChREBP. High glucose or xylitol challenge *ex vivo* induced the α1 and α2 catalytic subunits (*Prkaa1,2*), in hepatocytes from control mice but not from the GKA treated mice which showed improved ATP homeostasis (24). The attenuated induction of *Prkaa1,2* in hepatocytes from GKA-treated mice which have improved ATP homeostasis is shared by some (*Gckr, Fasn*) but not by other (*G6pc*) ChREBP target genes (24). This indicates a hierarchy of mechanisms resulting in activation of ChREBP target genes whereby some genes like *Prkaa1,2, Gckr*, and *Fasn* show attenuated induction in conditions of chronic ChREBP activation in association with improved ATP homeostasis whereas others like *G6pc* and ChREBP-β show sustained induction (24). The underlying mechanisms remain to be resolved. However, these studies provide support for drugs that activate ChREBP as a potential strategy for improving ATP homeostasis in non-alcoholic fatty liver disease.

## Perspectives

In the past 20 years since the discovery of ChREBP, a major focus has been on *Pklr* and *G6pc* and enzymes of fructose metabolism, lipid synthesis and secretion. The role of these genes in models of ChREBP activation or deletion has been well replicated. Nonetheless differences are noted between diverse models in the relative prominence of induction of *Pklr* versus *G6pc* as commented on elsewhere (23, 55). These widely studied ChREBP target genes are only a small fraction of the > 5000 candidate genes identified in mouse liver by genome wide analysis (8). The evidence implicating a role for the AMPK catalytic subunits *Prkaa1,2* (24) is of particular interest because it provides a mechanistic link between the attenuated induction by high substrate challenge in hepatocytes *ex vivo* of some (e.g. *Gckr, Fasn, Prkaa1,2*) but not other (*G6pc*) ChREBP target genes in models of varying resilience of ATP homeostasis. This indicates that the vulnerability or resilience of hepatocytes to maintaining ATP is a major determinant of the ChREBP target genes that are induced by a substrate challenge, and furthermore that the raised phosphate ester level is not the best marker. In this context a better understanding of ChREBP function may emerge from experimental models of mild versus severe metabolic stress, where different target genes of ChREBP may have greater or lesser roles.

## Author Contributions

Writing—original draft preparation, LA. Writing—review and editing, LA, SC, and BF. Funding acquisition, LA. All authors contributed to the article and approved the submitted version.

## Funding

Work in the authors’ laboratory is funded by the Medical Research Council, UK (MR/P002854/1).

## Conflict of Interest

The authors declare that the research was conducted in the absence of any commercial or financial relationships that could be construed as a potential conflict of interest.
